# Chorea-predominant SCA5 mimicking Huntington's disease: A case report with a novel *SPTBN2* variant

**DOI:** 10.1016/j.prdoa.2026.100501

**Published:** 2026-09-17

**Authors:** Peng Lei, Ying Chi, Jie Xia, Tao Wang, Ling Zhong

**Affiliations:** a Department of Neurology, Yichang Central People's Hospital, Affiliated Central People's Hospital, China Three Gorges University, Yichang, China; b Department of Neurology, Affiliated Renhe Hospital, China Three Gorges University, Yichang, China

**Keywords:** Chorea, SCA5, *SPTBN2*, Heterozygous variant, Huntington disease mimic

## Abstract

**Background:**

Chorea has diverse etiologies, with Huntington's disease (HD) being the most common genetic cause. While spinocerebellar ataxias (SCAs) rarely present with chorea, SCA5 is typically regarded as a “pure” cerebellar syndrome. To our knowledge, chorea has not been previously reported as the predominant manifestation of SCA5.

**Case presentation:**

A 51-year-old male presented with progressive dysarthria and generalized chorea. Family history suggested autosomal dominant inheritance. Cranial MRI revealed cerebellar atrophy and frontal-horn enlargement. Initially misdiagnosed with HD, he received olanzapine, achieving partial relief. Whole-exome sequencing (WES) subsequently identified a novel heterozygous missense variant in *SPTBN2*(c.2531G > A, p.Arg844Gln), revising the diagnosis to SCA5. At the 9-month follow-up, chorea was significantly improved.

**Conclusion:**

This case demonstrates that *SPTBN2*-related SCA5 can manifest as a chorea-predominant phenotype, easily misdiagnosed as HD. The identification of the novel c.2531G > A variant expands the phenotypic and mutational spectrum of SCA5. It underscores that WES should be considered early in autosomal dominant chorea with frontal-horn enlargement, particularly when cerebellar signs are subtle, to prevent diagnostic errors.

## 1 Introduction

Chorea is defined as an involuntary, rapid, irregular, and unpredictable movement disorder that flows from one body part to another. These dance-like movements can significantly interfere with daily activities [1]. The core pathophysiological hallmark of chorea is dysfunction of the basal ganglia [2]. The etiologies of chorea are broadly classified into non-genetic and genetic causes. Non-genetic etiologies are diverse, encompassing vascular, drug-induced, toxic, metabolic, autoimmune, paraneoplastic, infectious, and structural causes [1], [3], [4]. Genetic etiologies encompass a wide spectrum of conditions, including Huntington's disease (HD), neuroacanthocytosis, Huntington's disease-like syndromes (HDL), childhood-onset chorea, paroxysmal dyskinesias, and inherited disorders with brain metal accumulation. Other hereditary neurological diseases may also present with chorea [5]. Among these, Huntington's disease remains the most prevalent genetic cause. However, spinocerebellar ataxias (SCAs) have also been reported as rare causes of chorea [5].

SCAs refer to a genetically and clinically heterogeneous group of autosomal dominant neurodegenerative disorders. The clinical hallmark is progressive cerebellar syndrome, characterized by gait and limb ataxia, dysarthria, and oculomotor disturbances. Intriguingly, specific SCA subtypes often extend beyond that of pure cerebellar involvement, exhibiting additional extracerebellar manifestations such as pyramidal signs, extrapyramidal symptoms, ophthalmoplegia, and cognitive impairment [6]. The global prevalence of SCAs is estimated to be approximately 3–5 per 100,000 individuals [7]. To date, over 50 SCA subtypes have been identified. SCA3 is the most prevalent worldwide [8], followed by SCA2, whereas other subtypes are relatively rare. Chorea has been documented in several subtypes, including SCA2, SCA3, SCA6, SCA7, SCA8, SCA12, SCA14, SCA17, SCA27, SCA48, and dentatorubral-pallidoluysian atrophy (DRPLA), with SCA17 being the most commonly associated subtype [5], [6], [9]. Notably, chorea has not been previously reported as a manifestation of SCA5. Herein, we describe a 51-year-old male patient who presented with chorea as the predominant clinical feature. WES confirmed the diagnosis of SCA5, revealing a novel heterozygous missense variant in the *SPTBN2* (c.2531G > A, p.Arg844Gln), which has not been documented in previous literature.

### 1.1 Case presentation

**Fig. 1:**
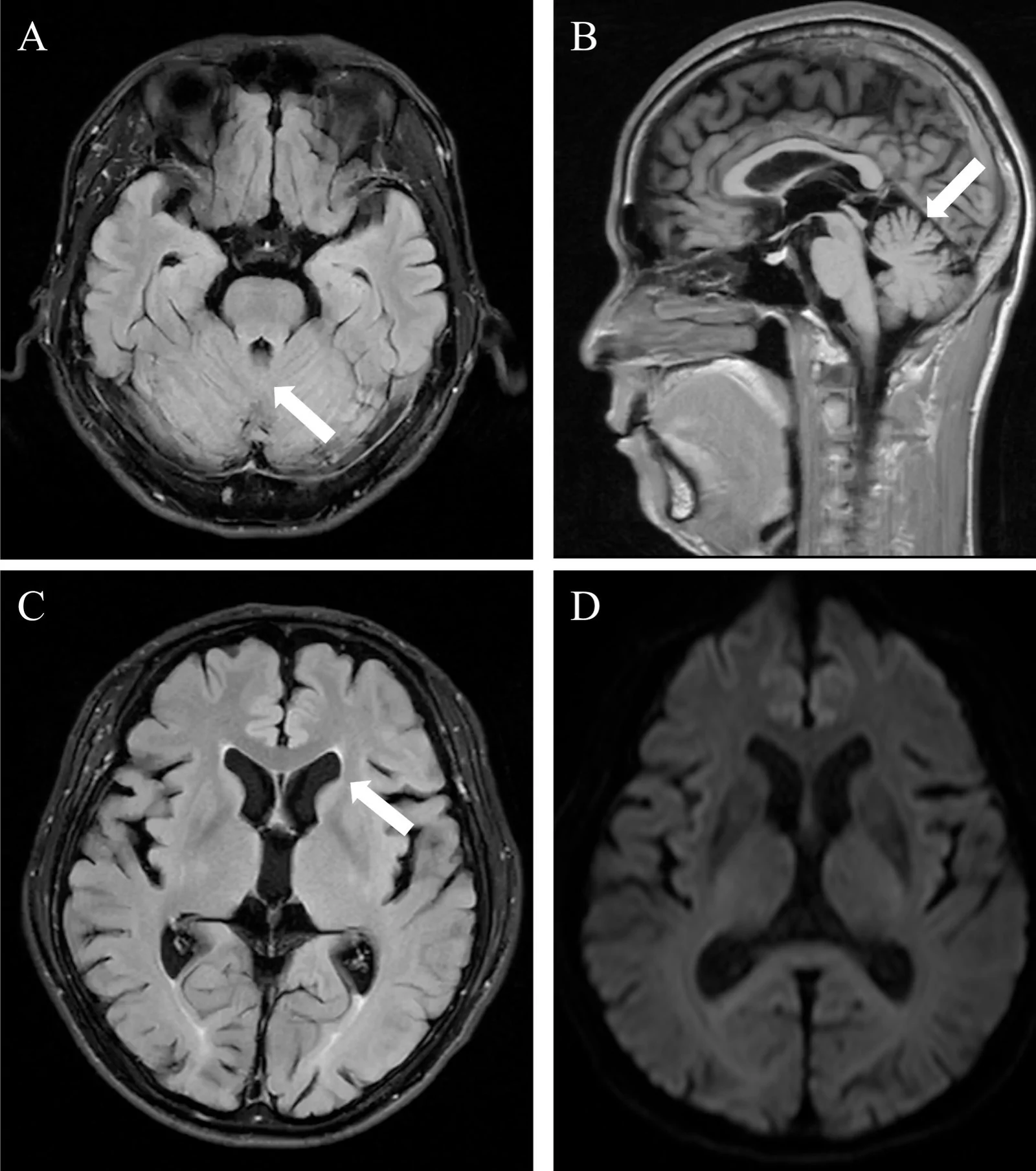
Serial brain MRI findings. Panels A–C: Scans performed at a referring tertiary hospital three months prior to admission. Panel A: Axial FLAIR image showing mild atrophy of the cerebellar vermis (arrow). Panel B: Sagittal T1-weighted image demonstrating mild cerebellar cortical atrophy (arrow). Panel C: Axial FLAIR image revealing enlargement of the frontal horns of the lateral ventricles (arrow). Panel D: Axial Diffusion-Weighted Imaging (DWI) acquired on the day of admission, showing no abnormal high signal intensity.

**Fig. 2:**
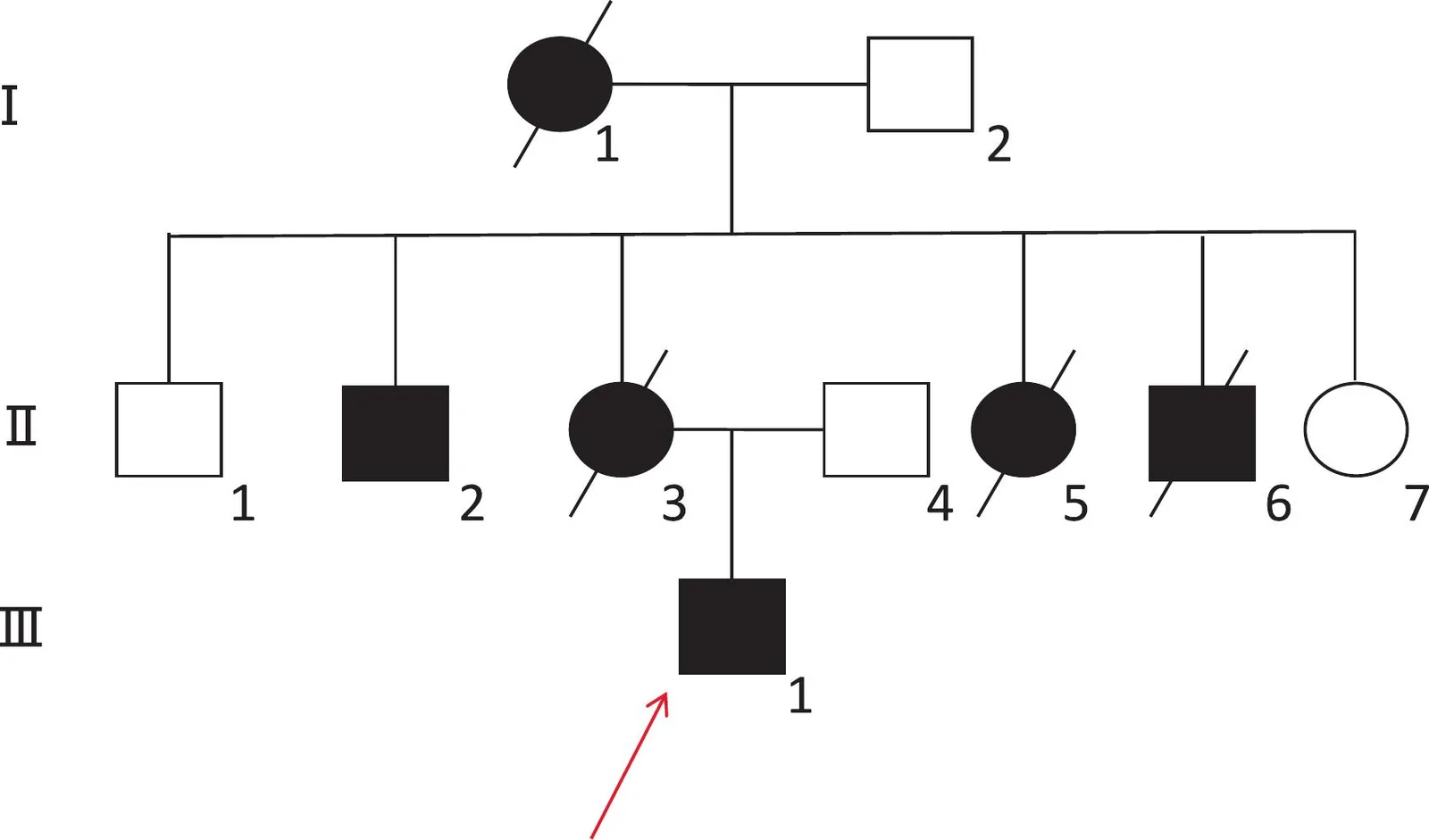
Pedigree: autosomal dominant transmission of involuntary movements. The pedigree spans three generations and demonstrates an autosomal dominant (AD) inheritance pattern. (symbols: ■ denotes affected males; ● denotes affected females; □ and ○ denote unaffected males and females, respectively. Deceased individuals are indicated by a diagonal slash (/) through the symbol. The arrow (→) indicates the proband (the 51-year-old male patient).

A 51-year-old male was admitted to our hospital on June 30, 2025, complaining of “slurred speech for 6 months and involuntary movements of the head, hands, and feet for 3 months.” The patient developed dysarthria insidiously 6 months prior, with no obvious precipitating factors. Three months ago, he began experiencing involuntary movements of the head, hands, and feet. Notably, there was no history of hallucinations, dementia, tremor, anxiety, depression, dizziness, headache, or insomnia. He remained fully ambulatory without assistance. He previously visited a local tertiary hospital, where cranial magnetic resonance imaging (MRI) revealed mild cerebellar cortical atrophy and enlargement of the frontal horns of the lateral ventricles (Fig. 1A-C). No specific treatment was administered at that time. Due to the progressive exacerbation of involuntary movements severely impairing his daily life, he sought further evaluation at our hospital. His past medical history was unremarkable, but family history was significant: his maternal grandmother, mother, maternal uncle, and maternal aunt all exhibited similar involuntary movements, consistent with an autosomal dominant inheritance pattern (Fig. 2, Table 1). Neurological examination revealed the following: Mental Status: Higher cortical functions were intact. Cranial Nerves: Mild dysarthria was noted. Eye movements were full, with no nystagmus. Motor System: Muscle strength was grade 5/5 in all four limbs. Tendon reflexes were symmetrical and normal. The plantar response (Babinski sign) was negative bilaterally. Cerebellum: Finger-to-nose and heel-to-shin tests were performed accurately. The Romberg sign was negative. However, he exhibited a slightly broad-based gait and was unable to perform tandem walking. Sensory System: Sensory examination was normal. Movement Disorder: Prominent choreiform movements were observed in the head, hands, and feet. Comprehensive laboratory investigations were performed to rule out acquired causes of chorea. Routine blood work, including complete blood count, liver and renal function, electrolytes, coagulation profile, lipid profile, and blood glucose (fasting and HbA1c), were all within normal limits. Tumor markers, creatine kinase (CK) and its isoenzymes, C-reactive protein (CRP), rheumatoid factor, antistreptolysin O (ASO), immunoglobulin G4 (IgG4), anti-neutrophil cytoplasmic antibodies (ANCA), extractable nuclear antigen (ENA) antibodies, and viral serology (hepatitis B surface antigen, hepatitis C antibody, syphilis, and HIV) were also unremarkable. Notably, serum ceruloplasmin was normal, excluding Wilson's disease. Serum protein electrophoresis revealed no monoclonal protein (M-protein). However, thyroid-stimulating hormone (TSH) was slightly decreased at 0.45 uIU/mL (reference range: 0.55–4.78 uIU/mL), while free triiodothyronine (FT3) and free thyroxine (FT4) remained normal.Electroencephalography (EEG) showed mildly increased fast-wave activity. Diffusion-weighted imaging (DWI) of the brain revealed no acute ischemic lesions or abnormal signals (Fig. 1D). A retrospective review of our hospital's electronic medical record (EMR) system revealed a relevant admission history. On December 10, 2023, the patient was hospitalized in the Department of Gastroenterology for intractable vomiting. Two days later, on December 12, the Neurology Department was consulted due to reported “abnormal mental behavior.” Notably, upon consultation, the consulting neurologist documented that the vomiting and psychiatric symptoms had completely resolved spontaneously. The physical examination at that time was significant only for mild involuntary movements, with all other neurological examinations being normal. The episode was preliminarily attributed to an anxiety state, and a referral to a psychiatric hospital was suggested. Clinical Reasoning: 1. Localization: The patient presented with hyperkinetic movements of the head, hands, and feet, indicating dysfunction of the basal ganglia. Additionally, the presence of mild dysarthria, broad-based gait, and inability to perform tandem walking localized the pathology to the cerebellum. 2. Qualitative Analysis: Given the middle age of onset, chronic progressive course, and significant family history suggestive of autosomal dominant inheritance, a genetic etiology was strongly suspected. 3. Initial Diagnosis and Management: Cranial MRI revealed cerebral atrophy with enlargement of the frontal horns, supporting the clinical suspicion of HD. Consequently, the patient was initially diagnosed with HD and discharged on oral olanzapine 5 mg qn, which partially alleviated the choreic symptoms. 4. Genetic Confirmation: The patient provided written informed consent to participate in the “Peking union Medical College Hospital-public welfare project for Rare Disease service Improvement” which subsidized free WES. WES performed 28 days later revealed a novel heterozygous missense variant in *SPTBN2* (NM_006946.4:c.2531G > A, p.Arg844Gln). This variant, located at chr11:g.66472216G > A, was absent from the literature and major population databases, including gnomAD v4 (AC = 0/730,947 exome alleles; AC = 0/76,215 genome alleles), ESP6500, and the 1000 Genomes Project. The absence of benign or likely benign entries in ClinVar supported an ultra-rare, non-polymorphic status, fulfilling the PM2_Supporting criterion per the ACMG/AMP 2015 framework [10], [11], [12], [13]. Although the clinical laboratory initially classified it as a variant of uncertain significance (VUS), integration with the clear autosomal dominant segregation pattern (affecting three successive generations on the maternal line) and the high evolutionary conservation of Arg844 within the spectrin-repeat domain shifted the weight of evidence in favor of a likely pathogenic (LP) interpretation. Concurrent *HTT* CAG repeat analysis was normal, excluding Huntington's disease and supporting the revised diagnosis of *SPTBN2*-related SCA5. (See Table 1.)

**Table 1:** Clinical characteristics of affected individuals in the SCA5 family.

individual	Relationship to proband	Age at onset(y)	Age at death(y)	Primary clinical manifestations
I-1	Maternal grandmother	39	65	Chorea involving the head, hands, and feet, along with progressive ataxia
II-2	Maternal uncle (eldest)	58	Alive	Chorea involving the head, hands, and feet, along with progressive ataxia
II-3	Mother	40	69	Chorea involving the head, hands, and feet, along with progressive ataxia
II-5	Maternal aunt (younger)	50	58	Chorea involving the head, hands, and feet, along with progressive ataxia
II-6	Maternal uncle (youngest)	50	58	Chorea involving the head, hands, and feet, along with progressive ataxia
III-1	Proband	50	Alive	Chorea involving the head, hands, and feet, along with progressive ataxia

Footnote: Abbreviations: y, years. “Alive” indicates the individual was still living at the time of last follow-up. Clinical data for affected relatives were obtained from medical history and previous medical records.The proband (III-1) is described in detail in the Case Presentation section. Notably, the only other living affected family member (II-2) declined further clinical evaluation, genetic testing, and neuroimaging.

At the 9-month follow-up, the patient returned to our neurology clinic. Remarkably, the choreiform movements of the head, hands, and feet had significantly improved (see Supplementary Video S1). However, the cerebellar signs—including mild dysarthria, broad-based gait, and inability to perform tandem walking—remained stable without noticeable change.

## 2 Discussion

This study reports a novel heterozygous missense variant in the *SPTBN2* (c.2531G > A, p.Arg844Gln) in a patient with SCA5 who presented primarily with chorea. Notably, this is the first documented case demonstrating that *SPTBN2*-related SCA5 can manifest as a hyperkinetic movement disorder rather than a “pure” cerebellar syndrome. These results broaden both the phenotypic and mutational spectrum of SCA5.

SCAs constitute a group of autosomal dominant neurodegenerative disorders. Pathologically, SCAs primarily affect the cerebellum, typically characterized by the loss of Purkinje cells, but they can also involve the spinal cord, brainstem, basal ganglia, and peripheral nerves [6]. SCAs exhibit remarkable genetic and clinical heterogeneity. The genetic etiologies of SCAs are diverse, including: (1) Polyglutamine (polyQ) diseases: Caused by the abnormal expansion of CAG trinucleotide repeat sequences within the coding regions of causative genes. This category includes eight disorders: SCA1, SCA2, SCA3, SCA6, SCA7, SCA17, SCA51, and dentatorubral-pallidoluysian atrophy (DRPLA). (2) Non-coding repeat expansions: Caused by abnormal expansions of tri−/multi-nucleotide repeat sequences in non-coding regions of the genes. Examples include SCA8, SCA10, SCA12, SCA27B, SCA31, SCA36, SCA37, and cerebellar ataxia with neuropathy and vestibular areflexia syndrome (CANVAS). (3) Other mutations: Including point mutations, insertions, deletions, and others. Representative subtypes include SCA5, SCA13, SCA14, SCA15, SCA19, and SCA28 [14]. Due to the high degree of clinical and genetic heterogeneity among SCAs, Harding proposed a clinical classification for autosomal dominant cerebellar ataxia (ADCA) in 1982 [15]. This classification system divides ADCAs into three distinct types:Type I (ADCA I): Characterized by progressive cerebellar ataxia combined with extracerebellar signs, such as pyramidal tract signs, extrapyramidal symptoms, and amyotrophy. This is the largest subgroup, encompassing SCA1–4, 8, 10, 12–14, 15, 17–22, 25, 27, 28, 31, 32, 34–38, 42–44, 46, 47, DNMT1, and DRPLA. Type II (ADCA II): Defined by the combination of cerebellar ataxia and retinal pigment degeneration. This type exclusively includes SCA7. Type III (ADCA III): Also known as “pure” cerebellar ataxia, presenting solely with cerebellar signs without significant extracerebellar involvement. This group includes SCA5, 6, 11, 23, 26, 30, 37, 41, and 45 [16], [17], [18], [19], [20]. However, with advancements in molecular diagnostics, accumulating evidence has challenged the rigid boundaries of Harding's ADCA classification. For instance, SCA6—traditionally classified as a “pure” cerebellar ataxia (ADCA III)—has increasingly been reported to present with Huntington's disease-like phenotypes, including chorea and psychiatric disturbances [9]. Similarly, our case defies the conventional classification of SCA5. While the patient exhibited mild cerebellar signs, the predominant manifestation was severe chorea.

SCA5 is a rare subtype of autosomal dominant cerebellar ataxia. It typically manifests in adulthood, with an average age of onset ranging from 30 to 40 years [21]. The clinical phenotype is traditionally characterized by progressive gait and limb ataxia, truncal instability, and dysarthria. Consistent with its classification as a relatively “pure” cerebellar disorder, SCA5 rarely involves the basal ganglia, brainstem, or spinal cord. Neuroimaging typically reveals cerebellar cortical atrophy, while the cerebral hemispheres, basal ganglia, brainstem, and cerebellar tonsils are usually spared. Pathological studies have corroborated these findings, demonstrating marked cerebellar cortical atrophy, loss of Purkinje cells, and thinning of the molecular layer [21]. SCA5 is caused by mutations in the *SPTBN2*, which is located on chromosome 11q13.2. This gene encodes β-III spectrin, a 2390-amino-acid cytoskeletal protein that is predominantly expressed in cerebellar Purkinje cells [21], [22]. Functionally, *SPTBN2* mutations disrupt neuronal axonal transport [23]. To understand the mechanistic basis for SCA5 phenocopying HD, we compared the downstream pathways affected by *SPTBN2* and *HTT* mutations. The wild-type huntingtin protein (HTT) regulates neurotrophic support and neuronal survival by enhancing the microtubule-dependent vesicular transport of brain-derived neurotrophic factor (BDNF). Conversely, the expanded polyglutamine tract (polyQ) in mutant HTT disrupts this transport machinery. This transport defect leads to a critical loss of BDNF signaling and subsequent neuronal toxicity, which is a central event in HD pathogenesis [24]. Clinically, HD is characterized by the triad of involuntary chorea, psychiatric disturbances, and cognitive decline [25]. Neuroimaging consistently reveals progressive cerebral atrophy, notably with caudate nucleus head atrophy and accompanying enlargement of the frontal horns of the lateral ventricles [1]. In this case, the patient presented predominantly with chorea and radiological evidence of cerebral atrophy with enlargement of the frontal horns. Combined with a positive family history of autosomal dominant inheritance, these features closely mimicked HD, leading to the initial misdiagnosis. However, WES identified a novel heterozygous missense variant in the *SPTBN2* (c.2531G > A, p.Arg844Gln), which revised the diagnosis to SCA5. We hypothesize that the substitution of arginine by glutamine at codon 844 disrupts the structural conformation and functional integrity of β-III spectrin. This perturbation may impair neuronal axonal transport, causing selective neurodegeneration in both the cerebellum and the basal ganglia. Consequently, the dual involvement of these circuits manifests as a novel phenotype combining chorea with mild cerebellar ataxia. Further functional studies are warranted to elucidate the precise pathogenic mechanisms underlying this specific mutation. Currently, no disease-modifying therapies exist for SCAs; management relies primarily on symptomatic and supportive care [14]. In this case, we prescribed olanzapine to control the patient's choreic movements. At the 9-month follow-up, the chorea had significantly improved, whereas the cerebellar ataxia remained stable without noticeable change.

Regrettably, we did not document the patient's baseline motor status on video during the initial visit. Furthermore, we were unable to obtain consent from other affected family members for WES, which limited our ability to perform co-segregation analysis and tempered the genetic robustness of our conclusions. Despite these limitations, our findings provide preliminary evidence that *SPTBN2*mutations can manifest as chorea-predominant SCA5.

## 3 Limitations

This study has limitations. The most significant is the lack of molecular confirmation of the *SPTBN2* variant in other affected family members. While the variant co-segregates perfectly with the disease phenotype across three generations, suggesting a causal relationship, we were unable to perform genetic co-segregation analysis due to the unavailability of DNA samples from deceased relatives and the declined participation of the only surviving affected family member (II-2), who also refused neuroimaging and detailed clinical evaluation. Furthermore, the absence of functional assays to validate the pathogenicity of the novel c.2531G > A variant limits our ability to establish a definitive causal link. Future studies involving functional validation or the eventual acquisition of familial DNA samples are warranted to confirm these findings.

## 4 Conclusions

This study identifies a novel *SPTBN2* variant (c.2531G > A, p.Arg844Gln) as a cause of SCA5 presenting with chorea as the predominant feature. Clinicians should consider SCA5 in the differential diagnosis of adult-onset chorea with mild cerebellar signs and characteristic imaging findings. Given the significant phenotypic overlap with HD, WES is indispensable for an accurate diagnosis and for preventing misdiagnosis. Functional validation of the p.Arg844Gln variant is warranted to confirm its pathogenic role.

The following are the supplementary data related to this article.

Supplementary Video S1 Clinical examination at the 8-month follow-up. The video demonstrates the patient's motor status while receiving olanzapine therapy. It highlights the significant improvement in choreiform movements affecting the head, hands, and feet compared to the initial presentation.

## CRediT authorship contribution statement

**Peng Lei:** Writing – original draft, Formal analysis, Data curation. **Ying Chi:** Writing – review & editing, Methodology, Formal analysis. **Jie Xia:** Writing – review & editing, Methodology, Funding acquisition, Formal analysis. **Tao Wang:** Writing – review & editing, Methodology, Formal analysis. **Ling Zhong:** Writing – original draft, Formal analysis, Data curation.

## Clinical trial number

Not applicable.

## Consent for publication

Written informed consent was obtained from the patient for the publication of this case report, including any accompanying images and supplementary video.

## Declaration of competing interest

The authors declare that they have no known competing financial interests or personal relationships that could have appeared to influence the work reported in this paper.

## Glossary

**List of abbreviations**
ADCA: Autosomal Dominant Cerebellar Ataxia
BDNF: Brain-Derived Neurotrophic Factor
HD: Huntington's Disease
HTT: Huntingtin
MRI: Magnetic Resonance Imaging
SCA: Spinocerebellar Ataxia
SPTBN2: Spectrin Beta, Non-Erythrocytic 2
WES: Whole-Exome Sequencing
